# Utilization of Mineral Wools as Alkali-Activated Material Precursor

**DOI:** 10.3390/ma9050312

**Published:** 2016-04-26

**Authors:** Juho Yliniemi, Paivo Kinnunen, Pasi Karinkanta, Mirja Illikainen

**Affiliations:** Fiber and Particle Engineering Research Unit, University of Oulu, Oulu 90014, Finland; paivo.kinnunen@oulu.fi (P.K.); pasi.karinkanta@oulu.fi (P.K.); mirja.illikainen@oulu.fi (M.I.)

**Keywords:** geopolymer, alkali activation, mineral wool, rock wool, glass wool, mmmf: man-made mineral fibre

## Abstract

Mineral wools are the most common insulation materials in buildings worldwide. However, mineral wool waste is often considered unrecyclable because of its fibrous nature and low density. In this paper, rock wool (RW) and glass wool (GW) were studied as alkali-activated material precursors without any additional co-binders. Both mineral wools were pulverized by a vibratory disc mill in order to remove the fibrous nature of the material. The pulverized mineral wools were then alkali-activated with a sodium aluminate solution. Compressive strengths of up to 30.0 MPa and 48.7 MPa were measured for RW and GW, respectively, with high flexural strengths measured for both (20.1 MPa for RW and 13.2 MPa for GW). The resulting alkali-activated matrix was a composite-type in which partly-dissolved fibers were dispersed. In addition to the amorphous material, sodium aluminate silicate hydroxide hydrate and magnesium aluminum hydroxide carbonate phases were identified in the alkali-activated RW samples. The only crystalline phase in the GW samples was sodium aluminum silicate. The results of this study show that mineral wool is a very promising raw material for alkali activation.

## 1. Introduction

Alkali-activated materials (AAM), also called geopolymers or inorganic polymers, have received a lot of attention lately because they have the potential to partly replace ordinary Portland cement (OPC) as a construction material. Studies have shown that AAMs have mechanical properties as good as, or even better than, OPC concrete [1]. In addition, other beneficial properties, such as fire resistance and their typically light weight, make it possible to use them for such purposes as constructing panels or making ceramics [2,3]. The increased interest in AAMs lies in the fact that they can be produced from waste materials, such as fly ash, mine tailings, and slags. In 2012, OPC production was estimated to account for 8% of global CO_2_ emissions [4], and government policies worldwide strongly encourage reducing OPC use. This explains the recent interest in finding alternative construction materials and binders.

Proper thermal insulation is needed throughout the world, because it is the most effective way to save energy used for heating and cooling buildings. Mineral wools—a general term for rock wool and glass wool—are the most common insulation materials in the world. They are produced at high temperatures by melting quartz sand, basalt, dolomite, and glass [5]. The molten mixture is fiberized by a high-speed spinning process. A small quantity of organic resin, typically phenol-formaldehyde, is used as an additive to bind the fibers together. As old buildings are torn down or renovated, a large amount of construction and demolition waste is generated, including the waste from mineral wools. Globally, 2.3 million tons of mineral wool waste was generated in 2010, and the amount is expected to rise to 2.5 million tons by the year 2020 [6]. Unfortunately, mineral wool is often unrecyclable [6]. The problems in recycling arise from the fibrous nature and low density of the material. Despite many attempts [7,8,9,10,11,12,13,14,15,16,17], the utilization of mineral wool waste in post-consumer production remains very low.

Both glass wool and rock wool have high contents of Si and X-ray amorphous mineralogy and, thus, have potential as AAM precursors. However, there has been to date only one preliminary study of utilizing rock wool as raw material for alkali activation [18]. Alkali activation of glass wool has not been previously studied. Mineral wools also have very consistent chemical and physical compositions, which make them even more attractive as raw materials for alkali activation.

This study experimented with the utilization of rock wool and glass wool as alkali-activation precursors. Both mineral wools were pulverized and activated with sodium aluminate. The effects of heat curing and organic resin removal on the physical and mineralogical properties of the mineral wools were analyzed.

## 2. Materials and Methods

Two types of virgin mineral wool were purchased from a hardware store for this study: rock wool (RW, Paroc, Paroc eXtra) and glass wool (GW, Saint Gobain, Isover KL37-100). The chemical composition of both mineral wools was determined with 4 kV X-ray fluorescence (XRF, PANalytical Omnian Axiosmax). For the XRF analyses, it was necessary to prepare melt-fused tablets from both mineral wool samples. The melt-fused tablets were produced from 1.5 g of sample melted at 1150 °C with 7.5 g of X-ray Flux Type 66:34 (66% Li_2_B_4_O_7_ and 34% LiBO_2_). The concentration of trace elements was determined by microwave-assisted wet digestion using a 3:1 mixture of HNO_3_ and HCl for 0.5 g of mineral wool and measured using an inductively coupled, plasma-optical emission spectrometer (ICP-OES, Thermo Electron IRIS Intrepid II XDL Duo, Thermo Scientific). The moisture content and loss-on-ignition at 350 and 525 °C were determined using thermo-gravimetric analysis equipment (PrepAsh, Precisa).

The RW consisted mainly of silicon, aluminum, calcium, magnesium, and iron. The GW contained 62.4 wt % of SiO_2_, but only 1.8 wt % of Al_2_O_3_ (Table 1). However, the Na_2_O content in the GW was high (16.8 wt %) in contrast to that of the RW (1.4 wt %). Table 2 shows the chemical compositions of the RW and GW determined using the ICP-OES method. The composition is in line with the XRF results (Table 1). However, the content of boron in the GW was significant, and it is possible that this was the result of the use of waste glass as a raw material in the GW manufacturing process.

The GW and RW samples were pulverized with RS200 vibratory disc mill (Retsch). Pulverizing was accomplished by milling mineral wool in 10 g patches for 30 s at a milling speed of 1500 min^−1^. This procedure was repeated several times to obtain a sufficient amount of pulverized mineral wool for further experiments and analyses.

The particle size distributions of the pulverized mineral wools were measured with a LS 13320 (Beckman Coulter), using the universal liquid module and applying the Fraunhofer model in calculations. Prior to the particle size measurements, the pulverized mineral wool was diluted with distilled water by using sodium pyrophosphate as a dispersing agent. The diluted sample was held in an ultrasonic bath for three minutes to ensure comprehensive dispersion before measurements were taken.

**Figure 1:**
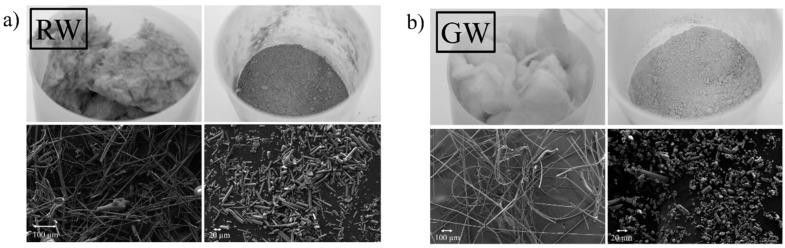
Photograph and FESEM images of (**a**) rock wool (RW); and (**b**) glass wool (GW) before and after grinding.

**Figure 2:**
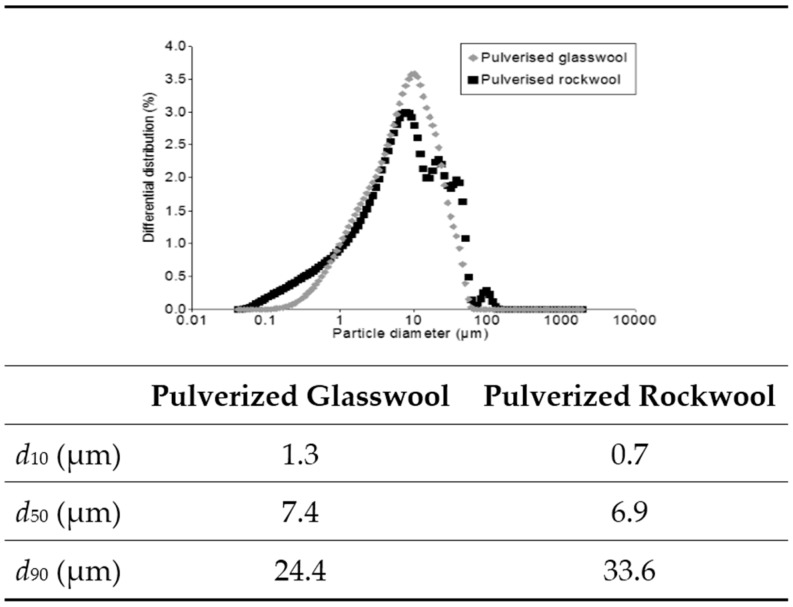
Particle size distributions of pulverized rock wool (RW) and glass wool (GW).

The appearances of the RW and GW before and after grinding are shown in Figure 1. Figure 2 presents the differential particle size distribution of the RW and GW after pulverization. The grinding was a very efficient means of pulverization, as the median size of both pulverized wools was approximately 7 microns, and 90% particles by volume (*d*_90_) was less than 34 microns in both cases (Figure 2). Although the pulverized wools had similar median particle sizes, there were slight differences between the differential particle size distributions (Figure 2).

The sodium aluminate solution was prepared by mixing sodium aluminate (Sigma-Aldrich, 13404), sodium hydroxide (VWR Merck, 1.06498.1000) and deionized water, using weight ratios of 14.3 wt %, 22.2 wt %, and 63.5 wt %, respectively.

The alkali-activated samples were prepared by adding the pulverized RW or GW to the sodium aluminate solution and mixing it in a mixer (ARE-250, Thinky Corporation) at 2000 rpm for 60 s. The mineral wools were added in 15–40 g portions to 63 g of sodium aluminate solution and mixed after each addition until the desired consistency was reached. In total, there were 140 g of RW and 150 g of GW in each sample. The mixture was vibrated in order to remove air bubbles and was then cast into molds, which were then sealed into a plastic bag until testing (28 days). Three batches were prepared from each type of mineral wool.

Table 3 shows the molar ratios of the alkali-activated samples. The molar ratios were set as close to 1:1:4:11 (Na_2_O:Al_2_O_3_:SiO_2_:H_2_O) [19] as possible. In order to obtain these ratios, both mineral wools had to be alkali-activated with sodium aluminate solution because of their lack of aluminum. However, sodium aluminate is not stable in liquid form at certain sodium-aluminum ratios [20], so optimal ratios were not achieved. In the GW samples in particular, the low quantity of aluminum yielded seemingly high Na_2_O, SiO_2_, and H_2_O ratios. Another thing to keep in mind is that a sodium aluminate solution can contain both 4-coordinated and 6-coordinated aluminum species depending on the [OH]/[Al] ratio [21], which can have an effect on the formation of aluminosilicate phases. In the molar ratio calculations, it was assumed that all elements dissolve from the mineral wools in the same proportions. Table 3 shows the molar ratios of CaO and MgO, because it is known that calcium and magnesium can participate in alkali activation [22,23,24].

After a total 28 days of curing, the Z100 testing machine (Zwick Roell) and TestXpert II software (Zwick Roell) were used to determine the unconfined compressive strength. Compressive force was increased at 2.4 kN/s until failure, and the maximum force was used to calculate the compressive strength. Flexural strength was measured with the Instron 5544 (Instron) (2 kN max force).

A Siemens 5000 X-ray diffractometer (Siemens) with CuKaα radiation (40 mA and 40 kV) and a graphite monochromator were used to identify the main crystalline phases of the pulverized mineral wools and prepared samples. The step interval, integration time, and angle interval used were 0.04°/step, 2.5 s/step, and 10°–60° 2θ, respectively. The International Center for Diffraction Data (ICDD) database was used for the identification of crystalline phases [25].

A Zeiss Ultra Plus field emission scanning electron microscope (FESEM, Zeiss) was used to analyze the original and pulverized mineral wools and the fracture surface of the prepared samples. The samples were attached to a sample holder on a carbon tape and coated with carbon. The acceleration voltage was 5 kV.

## 3. Results

### 3.1. Physical Properties of the Alkali-Activated Mineral Wools

The alkali-activated RW samples had an apparent density of approximately 2000 kg/m^3^, regardless of the curing conditions or resin removal (Table 4). The apparent density of samples GW1 and GW2 was roughly 1800 kg/m^3^, but the sample with organic resin removed had a density of 2037 kg/m^3^.

**Figure 3:**
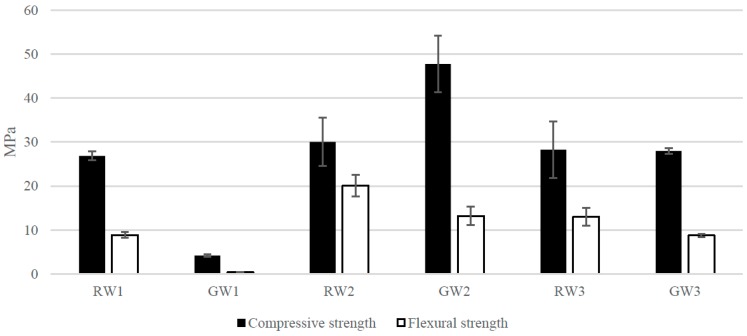
The compressive and flexural strength of each prepared sample. The bars show the average of at least three samples measured and the error represent the confidence interval for means at 95% confidence level.

**Figure A1:**
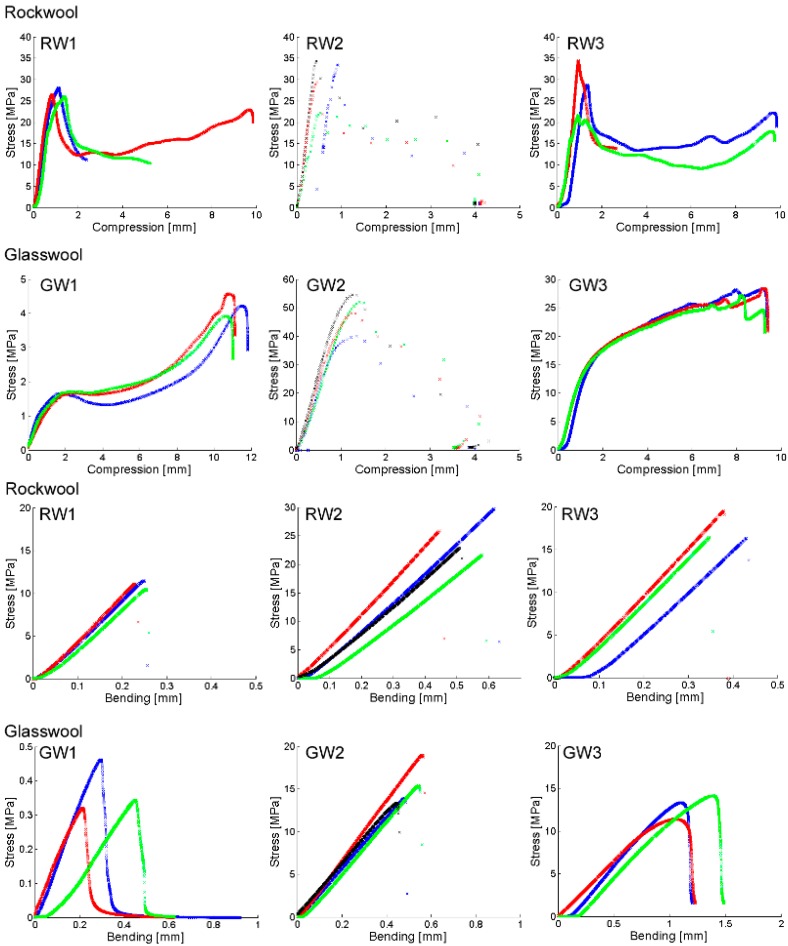
Stressing curves of studied mineral wool-based geopolymers under bending and compression. Each of the graphs show results of three or four different samples, which are indicated by different color.

The compressive and flexural strengths of the alkali-activated samples are presented in Figure 3. The stressing curves of each measurement are presented in the Appendix (Figure A1). The RW samples gained high compressive strength (~28 MPa), regardless of the curing temperature or presence of the resin. All samples, except GW1, had a very high flexural strength. The highest flexural strength (20.1 MPa) was in the heat-treated sample in which resin remained (RW2). The compressive and flexural strengths of the GW samples were low without the heat treatment (GW1). GW2 gained the highest compressive strength (48.7 MPa) of all of the prepared samples.

**Figure 4:**
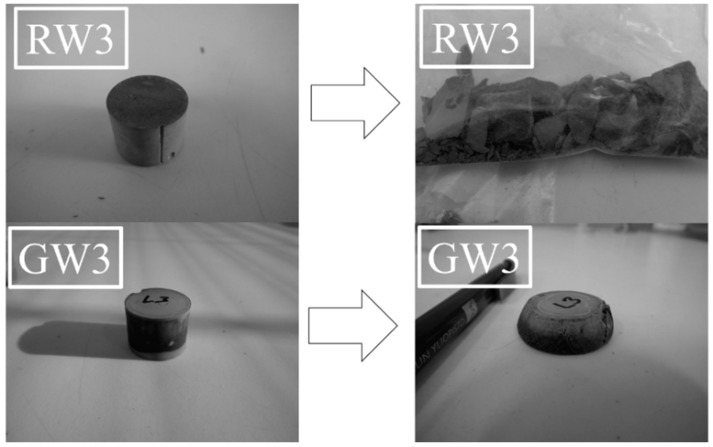
The deformation type of the alkali activated rock wool (RW) and glass wool (GW) samples.

It was observed that the RW samples broke into several pieces in the compressive strength tests, whereas the GW samples stayed in more or less one piece and were merely compressed (Figure 4). Especially peculiar behavior was observed for GW3, which was compressed by a mechanism called strain hardening (see Appendix Figure A1). In strain hardening, the material requires an ever-increasing amount of stress to continue straining [26]. The GW1 sample cured at room temperature was a bit moist even after 28 days, but all other GW and RW samples were dry. There was no sign of the formation of salts (*i.e.*, efflorescence) on the surface of the samples during the first four months of hardening.

### 3.2. FESEM Analysis

**Figure 5:**
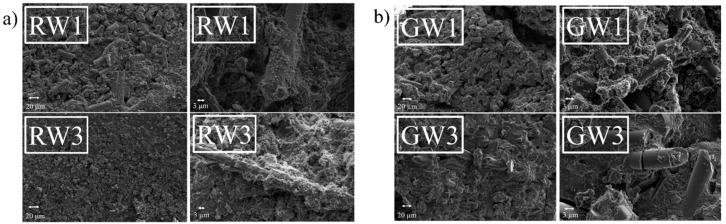
Secondary electron image of the fracture surface of (**a**) the rock wool (RW); and (**b**) glass wool (GW) geopolymer samples. On the left side of the images is a general look of the surface and on the right side of the images is a more detailed image showing the composite-type structure.

Figure 5 presents the typical look of the fracture surfaces of the alkali-activated rock wool (RW) and glass wool (GW) samples. On the right side of the images, a magnification of the partly-dissolved fibers is shown. The samples cured at room temperature had a significantly more porous structure than the heat-cured samples. The pieces of pulverized mineral wools can be observed to be bound together by the matrix.

### 3.3. XRD Analysis

**Figure 6:**
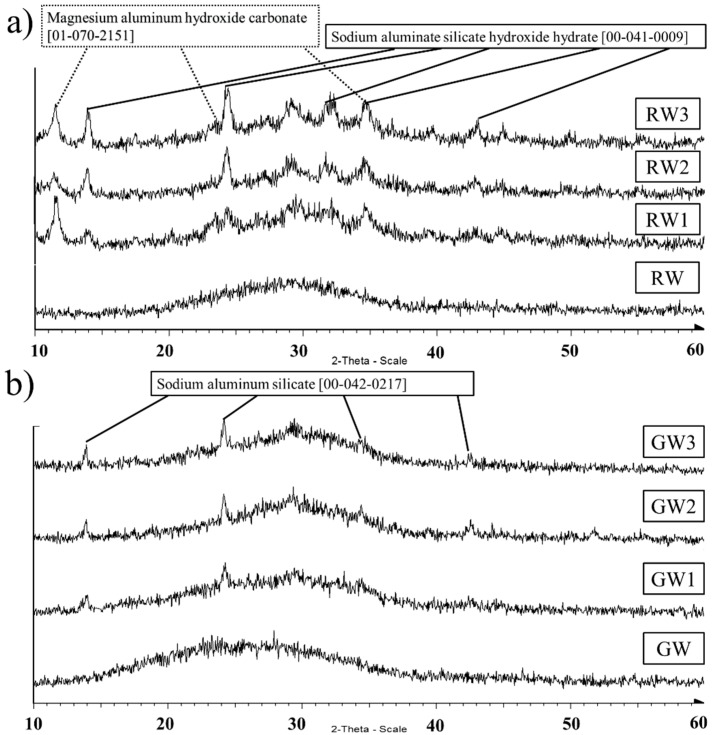
X-ray diffractograms of the (**a**) rock wool (RW); (**b**) glass wool (GW), and the alkali-activated samples.

Both mineral wools were completely X-ray amorphous (Figure 6). The amorphous hump was observed between 20° and 38° 2θ for RW, whereas it was wider for GW, starting from 14° and ending around 40° 2θ. The shift of the amorphous hump is considered to be an indicator of newly-formed nanocrystalline zeolites [27]. It can be observed that the shift of the amorphous hump is more notable for GW. This is mainly because the center of the hump in GW is around 25° 2θ before the alkali activation and 30° 2θ after the activation. For RW, the center of the hump is around 30° 2θ before alkali activation; thus, the shift is not as clearly observable.

In alkali-activated RW samples (RW1-3), sodium aluminate silicate hydroxide hydrate (00-041-0009) and magnesium aluminum hydroxide carbonate (01-070-2151) phases were found, in addition to the amorphous phase. A similar sodium aluminum silicate hydrate (NASH) phase was found in a previous study of RW alkali activation by sodium aluminate solution [18]. Additionally, Van Riessen *et al.* [28] found a NASH phase when coal fly ash was activated with a sodium aluminate solution. A sodium aluminate silicate (00-042-0217) phase was identified in GW1-3. A remark about the identification of these phases should be made. The identification was not absolute, because there were other similar aluminosilicate phases that had signals at the same 2θ positions. Also, the relatively wide signals indicate the presence of other similar type of phases than identified. However, the phase signals that fitted the best to the X-ray diffractograms were chosen and are presented here.

## 4. Discussion

The relationship between the molar ratios and physical properties of AAMs is often discussed. According to a study by Duxson *et al.* [19], the molar ratio of Si/Al should be around two and that of Na/Al should be one in order to complete the charge balancing of the negatively-charged tetrahedral aluminum centers. The amount of water should be as low as possible, because water is not usually considered to be part of the structure but acts merely as a medium in which the reactions occur and serves to maintain workability. However, recent studies [29,30,31] have shown that C-(N)-A-S-H gels can have bound water in the structure.

Regardless of the far-from-optimum molar ratios for GW, GW2 gained the highest compressive strength of all of the samples. Theoretically, Si-O-Si are stronger bonds than Si-O-Al or Al-O-Al bonds [32], and the very high Si/Al ratio of GW2 could explain its high compressive strength. However, boron, which was present in GW, has also been noticed to increase the strength of the AAMs [33,34]. One possibility is that silicon and aluminum do not dissolve at the same proportions, as they are present in the mineral wools and, thus, the calculated molar ratios would not represent the real molar ratios of the binder system. This note is supported by the aforementioned fact that Si-O-Si are stronger bonds than Si-O-Al or Al-O-Al bonds, which would result in aluminum dissolving in higher proportions than silicon.

One other difficulty in explaining the compressive strength differences using molar ratios arises from the XRD analysis, because this analysis shows only the crystalline phases, which may not be the main strength-increasing aluminosilicate structure. Instead, the newest phases are X-ray amorphous, and, thus, the chemical composition of the new nanocrystalline phases remains undetermined.

The main difference between the RW and GW, in addition to the aluminum content, was the high content of Ca and Mg in the RW. Both have been shown to be reactive in alkali activation [22,23,24], but only the formation of a new Mg phase was identified in the RW samples. The formation of a hydrotalcite-type magnesium phase shows that Mg from RW reacts with CO_2_, thus acting as a CO_2_ sorbent in the formation of these binders. A similar hydrotalcite-type phase has been observed in the alkali activation of blast furnace slag (BFS) [23]. In CaO-rich systems, the strongly alkaline activating solution can favor the pozzolanic reaction between CaO and SiO_2_, producing calcium silicate hydrate (CSH). If present, this phase can give broad peaks at about 30° and 50° 2θ. The former is clearly visible in the XRD traces (Figure 6) of the RW1, RW2, and RW3 samples and barely visible in those of the GW2 and GW3 samples (the CaO content of GW is lower than that of RW, but not negligible).

It has been shown that in low-Ca binder systems, heat treatment accelerates the hardening and increases early mechanical strength [35,36,37]. However, for high-Ca systems, heat curing has been observed to negatively affect the mechanical strength [38,39]. The severe drying shrinkage of the specimens with high-Ca type gels may be one reason for the lower strength. The phenomenon has been also explained by the fast calcium reactions, which are then accelerated by the elevated temperature, thus inhibiting further reactions (formation of secondary phases). However, for low-Ca binder systems, the binding of water into NASH gel is slow and/or weak; thus, heat curing speeds up the reaction (but only if the samples are sealed, as this prevents the evaporation of water). This could partly explain the high increase in compressive strength of the heat-cured GW samples and the negligible increase in strength with heat-cured RW samples.

The studies that have considered binder structure with a high or low calcium content have mostly used a (sodium) hydroxide or (sodium) silicate activator [22,40], and there are only a few studies in which sodium aluminate is the activator of choice [21,28,41,42]. Additionally, the high-Ca studies have been mostly conducted with BFS as a precursor, so the observations found in the literature may not be directly applicable with our binder system. However, both the BFS and mineral wools are manufactured in high temperatures and from similar precursors, so it might be appropriate to consider them as similar types of AAM precursors.

The presence of organic resin in the mineral wools had a positive effect on the strengths; thus, it is not necessary to remove it prior to the alkali activation. The reason for this is either that the organic resin physically strengthens the aluminosilicate matrix or that it reacts chemically with the other binder components. During the preparation of the samples, it was observed that the samples in which the organic resin was present had a very strong smell, but samples RW3 and GW3 were odorless. This indicated the success of organic resin removal in samples RW3 and GW3, but also signaled possible reactions with Na-Alu and organic resin in samples RW1, RW2, GW1, and GW2. The effect of the organic resin on these binder systems will be studied further.

The high flexural strength of the samples can be explained by their composite-type matrix, as shown in Figure 5. However, as the fibers are dissolved and chemically bound to the matrix, the long-term mechanical performance must be carefully determined, because if the mineral wool fibers will eventually dissolve fully, the matrix will consist only of aluminosilicate gel. The dissolution of mineral wool fibers could yield a better or worse mechanical strength depending on whether the formatting aluminosilicate structure is stronger or weaker than the fibers.

## 5. Conclusions

This study shows that mineral wools have great potential as precursors to alkali activation, because they have suitable chemical and mineralogical compositions. Heat curing and the presence of organic resin increased the mechanical properties. Maximum compressive strengths of 48.7 and 30.0 MPa were measured for GW and RW, respectively. The binder matrix consisted of aluminosilicate gel with partly-dissolved mineral wool fibers. The maximum flexural strength was 13.2 MPa for GW and 20.1 MPa for RW. The sodium aluminate silicate phase was the only crystalline phase in the GW samples. In RW samples, sodium aluminate silicate hydroxide hydrate and magnesium aluminum hydroxide carbonate were identified, in addition to the X-ray amorphous material. The apparent density of all the samples varied between 1750 and 2100 kg/m^3^. The results of this study show that high-strength AAMs can be obtained without any additional co-binders by alkali activating them with sodium aluminate solution.

## Figures and Tables

**Table 1:** The chemical composition, dry matter content, and loss-on-ignition of rock wool (RW) and glass wool (GW), determined by XRF and TGA.

Chemical Component	Rock Wool (RW)	Glass Wool (GW)
CaO	17.4	7.1
SiO_2_	40.4	62.4
Al_2_O_3_	15.8	1.8
Fe_2_O_3_	9.2	0.6
Na_2_O	1.4	16.8
K_2_O	0.4	0.9
MgO	12.6	2.2
P_2_O_5_	0.1	n.d
TiO_2_	0.8	n.d
SO_3_	n.d	0.9
Cl	n.d	0.1
Dry matter content (%)	100.0	100.0
Loss-on-ignition 350 °C (%)	1.8	5.0
Loss-on-ignition 525 °C (%)	2.4	5.1

Note: n.d represents “not detectable”.

**Table 2:** The chemical composition of rock wool (RW) and glass wool (GW), determined by ICP-OES.

Chemical Component	Rock Wool (RW)	Glass Wool (GW)
Ca, ICP (g/kg)	125	20.1
Si, ICP partial solution (g/kg)	0.4	0.4
Al, ICP (g/kg)	85.6	0.3
Fe, ICP (g/kg)	61.3	0.9
Na, ICP (g/kg)	10.4	68.2
K, ICP (g/kg)	3.7	3.7
Mg, ICP (g/kg)	75.8	3.9
P, ICP (g/kg)	0.3	<0.020
Ti, ICP (g/kg)	3.5	<0.050
S, ICP (g/kg)	0.1	3.8
Ba, ICP (g/kg)	0.2	1.3
Mn, ICP (g/kg)	1	0.5
As, ICP (mg/kg)	<3	<3
Cd, ICP (mg/kg)	<0.3	<0.3
Cr, ICP (mg/kg)	280	2.3
Cu, ICP (mg/kg)	34	8.8
Hg, CVAAS (mg/kg)	<0.04	<0.04
Ni, ICP (mg/kg)	49	1.8
Pb, ICP (mg/kg)	<3	3.7
Zn, ICP (mg/kg)	47	430
B, ICP (mg/kg)	8.9	6260
Be, ICP (mg/kg)	<1	<1
Co, ICP (mg/kg)	21	2
Mo, ICP (mg/kg)	<1	<1
Sb, ICP (mg/kg)	<3	<3
Se, ICP (mg/kg)	<3	<3
Sn, ICP (mg/kg)	<3	<3
V, ICP (mg/kg)	170	<2

Note: CVAAS represents “cold-vapor atomic absorption spectrometry”.

**Table 3:** The sample name, binder molar ratio composition, and curing temperature and time.

Sample Name	RW1	GW1	RW2	GW2	RW3	GW3
Mineral wool type	Pulverized rock wool	Pulverized glass wool	Pulverized rock wool	Pulverized glass wool	Pulverized and resin removed rock wool	Pulverized and resin removed glass wool
Na_2_O (mol)	0.7	7.6	0.7	7.6	0.7	7.5
Al_2_O_3_ (mol)	1.0	1.0	1.0	1.0	1.0	1.0
SiO_2_ (mol)	3.6	20.5	3.6	21.2	3.6	21.1
H_2_O (mol)	8.4	29.5	8.4	30.3	8.5	30.4
CaO (mol)	1.7	2.5	1.7	2.6	1.7	2.6
MgO (mol)	1.7	1.1	1.7	1.1	1.7	1.1
Curing (temp. and time)	28 days in 22 °C	28 days in 22 °C	4 days in 50 °C and then 24 days in 22 °C	4 days in 50 °C and then 24 days in 22 °C	4 days in 50 °C and then 24 days in 22 °C	4 days in 50 °C and then 24 days in 22 °C

**Table 4:** The apparent density of each prepared sample.

Sample Code	Density (kg/m^3^)
RW1	2093
GW1	1779
RW2	2003
GW2	1802
RW3	1956
GW3	2037
